# Both a Gauge and a Filter: Cognitive Modulations of Pupil Size

**DOI:** 10.3389/fneur.2018.01190

**Published:** 2019-01-22

**Authors:** R. Becket Ebitz, Tirin Moore

**Affiliations:** ^1^Department of Neuroscience and Center for Magnetic Resonance Research, University of Minnesota, Minneapolis, MN, United States; ^2^Department of Neurobiology, Stanford University School of Medicine, Stanford, CA, United States; ^3^Howard Hughes Medical Institute, Seattle, WA, United States

**Keywords:** pupil light response (PLR), pupil size, visual perception, attention, pupil light reflex, decision-making, exploration

## Abstract

Over 50 years of research have established that cognitive processes influence pupil size. This has led to the widespread use of pupil size as a peripheral measure of cortical processing in psychology and neuroscience. However, the function of cortical control over the pupil remains poorly understood. Why does visual attention change the pupil light reflex? Why do mental effort and surprise cause pupil dilation? Here, we consider these functional questions as we review and synthesize two literatures on cognitive effects on the pupil: how cognition affects pupil light response and how cognition affects pupil size under constant luminance. We propose that cognition may have co-opted control of the pupil in order to filter incoming visual information to optimize it for particular goals. This could complement other cortical mechanisms through which cognition shapes visual perception.

## Introduction

The first filter through which the visual world passes is the pupil. We use the word “filter” because the pupil is not a passive window unto the world. The pupil is constantly changing size as the musculature of the iris constricts and dilates. These adjustments have consequences for the amount of light that hits the retina, but also for the quality of our percepts of the visual world—how we see the world and, by extension, interact with it.

We can read a remarkable amount of information about people's cognitive processing through their pupils. For example, the pupil dilates in response to attractive social partners (1, 2). This is such an important interpersonal signal that women in the Middle Ages used belladonna (a dangerous poison) to dilate their pupils in order to attract partners. Of course, belladonna would also have consequences for the user's perception. This is because it produces pupil dilation, which increases optical aberrations (3–6). By scattering photons of light, these aberrations add positional noise in terms of where light hits the retina, thereby reducing high spatial frequency information and effectively rendering the visual world in softer focus. In photography, soft focus is frequently used to produce a youthful and romantic glow (7, 8). Thus, it is possible then that pupil dilation signals attraction to other humans and amplifies attraction by rendering social partners in a softer, more attractive focus.

Attraction is not the only mental process that influences the size of the pupil. Pupil size scales with mental effort (9, 10), surprise (11, 12), attention (13–15), and abstract goal states such as exploration (16–18). As tools for measuring pupil size become more readily available, pupil size is increasingly being used as a non-invasive peripheral index of cognitive processes. It is tempting to think of these modulations as simply a fortunate byproduct of a cognitive process of interest. However, it is also possible that cognitive modulations of the pupil have a function. They may be an adaptive motor response generated by that cognitive process. Just as attraction increases pupil dilation, which, in turn, may render a more attractive world, it is possible that cognition adjusts pupil size in order to produce specific changes in our visual percepts. There is certainly evidence that cognitive processes shape visual processing via other mechanisms. For example, there are rich descending projections from prefrontal to visual cortex which change information processing and visual representations (19). Cognition also controls where we position our fovea—that is, which points of the visual scene we acquire high spatial frequency information about (20–22). In both cases, cognition acts to enhance and emphasize visual features that are relevant to that cognitive process: it optimizes perception toward its own ends.

In this review, we first discuss possible functions of cognitive modulations of the pupil light response—a pupil reflex arc that is essential for light adaptation. Then, we apply this same functional framework to consider the effect that spontaneous or cognitive fluctuations in pupil size may have on visual perception. We build an intuition for these effects by briefly reviewing how the aperture was used to produce different qualities of images in early art photography. However, we caution that much additional work is necessary to determine the extent to which physiological changes in pupil size affect gaze and perception. Ultimately, the goal of this review is to highlight these open questions and identify next steps for research on the perceptual consequences of pupil size.

## Attention and the Pupil Light Response

**Figure 1:**
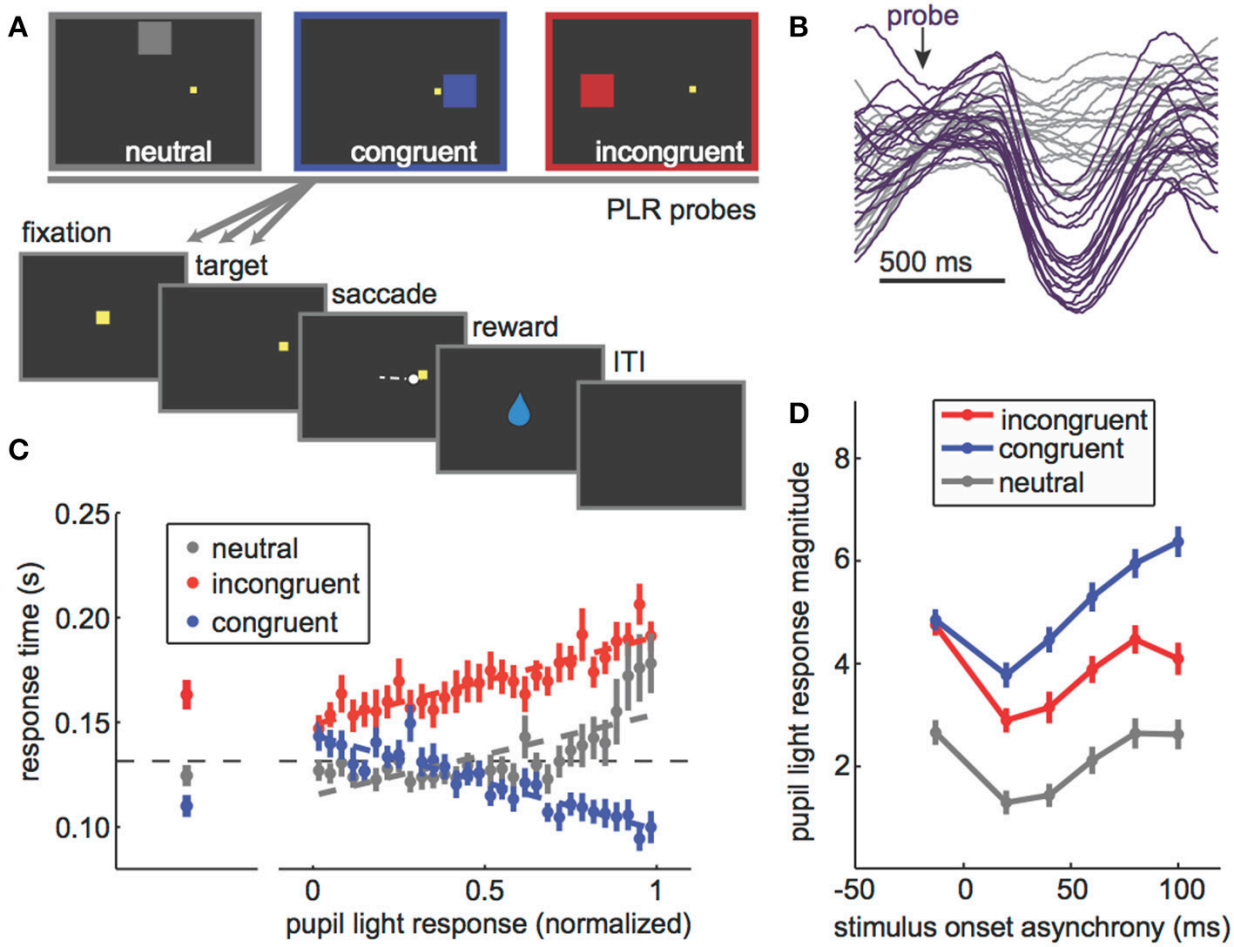
The PLR is correlated with stimulus attention and PLR magnitude can be used to probe the dynamics of visuospatial attention. **(A)** The PLR distraction task. In this task, PLR-evoking probes are presented in one of three locations relative to a rewarded target: above fixation, away from all possible target locations (“neutral”), on the same side as the rewarded target (“congruent”), or in the opposite hemifield from the rewarded target (“incongruent”). PLR probes were presented both before and at various latencies after target onset. **(B)** Some example pupil traces [data from Ebitz and Moore (28)] showing the characteristic light-evoked constriction after an evoking probe (purple) compared to sham-probe trials (gray). **(C)** Left: Response time effects of PLR probes in each location. Congruent probes sped response times, while incongruent ones slowed response time. Neutral probes had little impact on response time. Right: The evoked PLR strongly predicted the extent to which that probe would capture attention, as measured by response time effects of the probes. **(D)** PLR magnitude (bigger = more constriction) as a function of the timing of the PLR probe. If the probe was presented before the rewarded target (negative stimulus onset asynchrony), there was no difference between congruent and incongruent probes. All PLRs were suppressed to probes presented immediately after the rewarded target. Then, as monkeys began to prepare a saccade to the rewarded target, congruent probes PLRs (blue) were enhanced relative to both incongruent (red) and neutral (gray). Figures modified from Ebitz et al. (14) and Ebitz (29) under a Creative Commons Attribution license and with permission from copyright holders.

The pupillary light reflex (PLR; Figure 1B) is the first and most fundamental mechanism for light adaptation in the brain. When a focal or global luminance change occurs, the pupil constricts (23). This constriction is generally thought to serve a protective function, preventing photoreceptor fatigue and transient blindness when luminance increases (23). The PLR is mediated through a subcortical reflex pathway. Luminance information from the retina is relayed to the midbrain pretectal nucleus, which in turn projects to the Edinger-Westphal nucleus, which signals the pupillary sphincter to contract (23, 24). However, the subcortical reflex pathway is not the only pathway by which visual information can influence the pupil response to increasing luminance. For example, in the absence of direct retinal input to the pretectum—when the subcortical pathway is eliminated—a small pupillary light response can still be observed. Moreover, postgeniculate, cortical lesions can impair normal pupil light responses, though these effects are smaller than the consequences of eliminating the subcortical pathway (25–27). Thus, the pupil light reflex is only one small part of a larger PLR, some of which is mediated by cortical processing.

The existence of cortical influences raises the possibility that higher-order visual or even cognitive processes could shape the PLR. Indeed, there is empirical evidence for this view. For example, we know that the PLR is reduced during performance of a competing task (30), suggesting that it could be subject to resource limitations (31). Studies linking the PLR specifically to changes in visual processing go back at least as far as the 1940s. For example, in 1948, a binocular rivalry study examined the PLR evoked by illuminating one eye. They found that the PLR was larger when the visual input to that eye was dominating perception, compared to when it was not (32). This result was soon replicated (33) and other studies began finding that the PLR depended on visual processing in other ways. For example, a stimulus detection study found that the PLR was absent for probes reported as not seen (34) and a presaccadic processing study found that the likelihood of evoking a PLR was suppressed before a saccade (35)—following a similar time course as the presaccadic suppression of visual perception [also see (36)]. One unifying interpretation of these observations is the idea that the PLR is modulated by visual attention. This is because attention is strongly linked to ocular dominance (37, 38), target detection (39), and saccadic preparation (40–44).

However, it was not until much later that studies began to explicitly test the idea that the PLR is modulated by selective visual attention (13–15, 29, 45). For example, instructions to attend to a bright stimulus enhance the PLR to that stimulus, while instructions to attend away diminish PLR magnitude (13, 15). The PLR also tracks trial-by-trial variability in the selective attention paid to an evoking probe [Figures 1A,C; (14, 29)]. Preparing an oculomotor response to a probe location, which is known to recruit visual spatial attention (41, 43), also enhances the PLR to that probe (14, 29, 46, 47). Together, these results showed that the PLR scales with visual attention regardless of whether it is endogenously cued (13, 15), exogenously cued (14, 29, 48), and or recruited by saccadic preparation [Figure 1D; (14, 29, 46, 47)].

**Figure 2:**
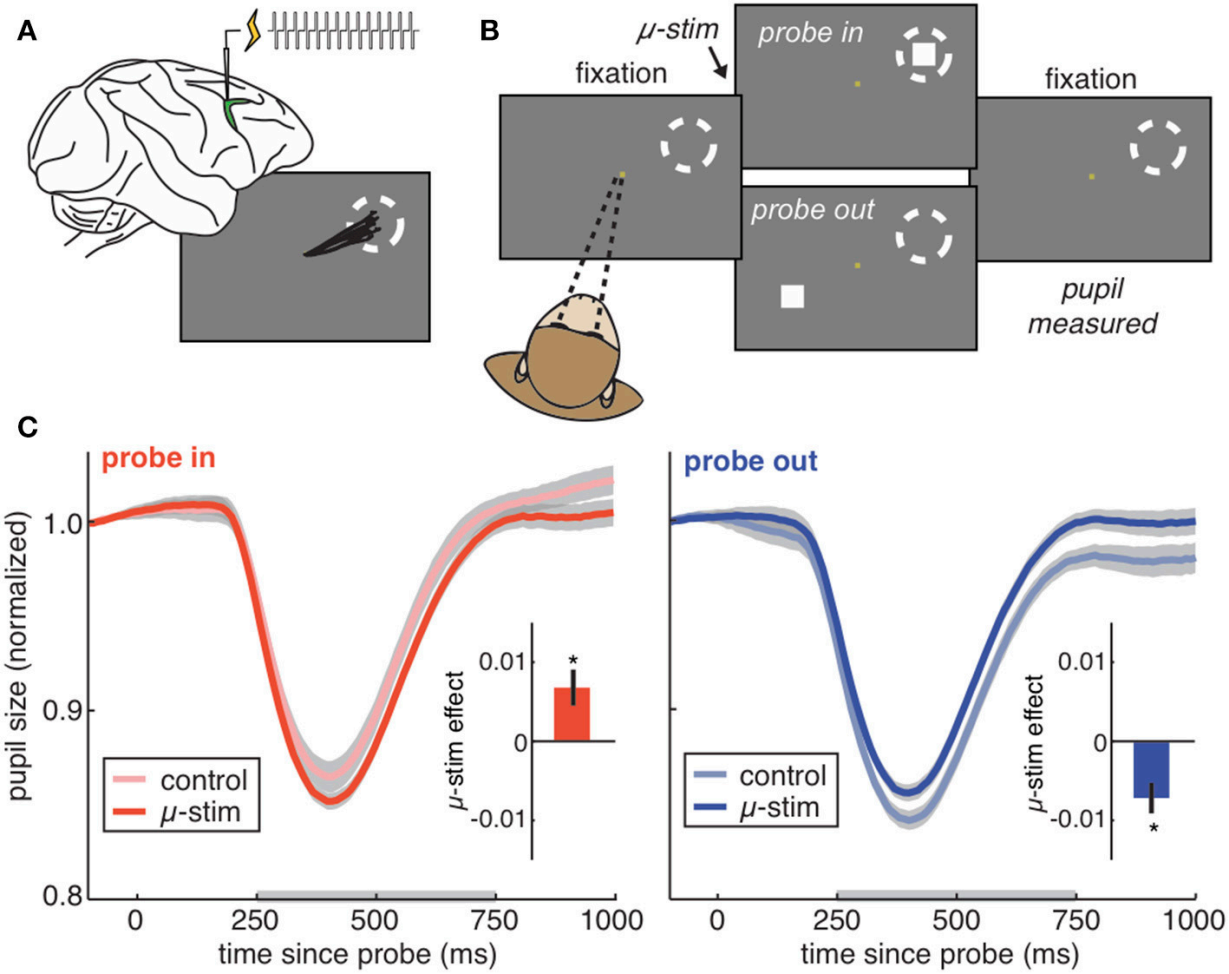
The PLR is bidirectionally modulated by cortical stimulation. **(A)** The frontal eye fields (green) are a part of prefrontal cortex responsible for directing gaze and attention. Injecting current into the frontal eye fields produces repeated saccades to the same location in space (the “response field,” dotted circle). Stimulating at very low currents (“microstimulation”) directs covert visual attention to the response field without moving the eye. **(B)** The PLR stimulation task. Rhesus monkeys hold fixation while PLR-evoking light probes are flashed on the screen. On some trials, microstimulation is delivered in order to direct covert visual attention to one of the two possible probe locations. **(C)** The pupil light response from an example session on trials where the probe was flashed in the stimulated response field (left), our outside of the stimulated field (right). Pale colors = no stimulation control trials. Saturated colors = stimulation was delivered. Inset) Difference between control and stimulation trials across all sessions. *p < 0.05. Figures modified from Ebitz and Moore (28), reproduced under a Creative Commons Attribution license.

It seems plausible that attention-related modulations of the PLR would require cortical control. But what is the source of this control? The pretectal nucleus receives input from the frontal eye field (FEF), an area within prefrontal cortex (49–51), and the lateral intraparietal cortex (52). These regions are causally implicated in selective attention (53, 54) and saccadic control (55–59). Moreover, we have previously found that microstimulation of the FEF is sufficient to bidrectionally modulate the gain of the PLR [Figure 2; (28)]. Thus, the PLR may be subject to prefrontal control by the same region causally responsible for shaping visual perception in the service of attention (19). Because of these studies, a strong argument can be made that the PLR is a valid, implicit, peripheral measure of selective visual attention. That is, (1) it is modulated by the same cognitive and presaccadic processes involved in selective visual attention (13–15, 29, 47, 48) and (2) it is modulated by the same neural perturbations that cause other correlates of selective visual attention, such as changes in extrastriate cortex (13, 28). Thus, the PLR is a powerful new way to measure selective attention in circumstances in which it was not previously possible (60). However, the use of the PLR as an implicit index of attention should not preclude the possibility that these modulations have a function.

### Possible Functions of Attentional Modulations of the Pupil Light Response

The first and most often cited function of the pupil light reflex is for light adaptation. Perhaps one function of the attentional modulations of the PLR is to allow light adaptation across saccades (14, 28, 46). We know that selective attention is an integral part of saccade planning (41, 43), so perhaps attentional modulations of the PLR play a presaccadic role. In natural vision, two sequential saccades may target regions that differ in luminance by several orders of magnitude (61). Anticipatory light adaptation could be useful because the pupil requires hundreds of milliseconds to constrict (62). Initializing this process before a saccade would give the pupil time to begin to constrict before a bright target is foveated—ensuring that the pupil is at least partially constricted before the retina is oriented toward a bright eccentric target. Indeed, one elegant study found that the luminance information at the target of an upcoming eye movement is integrated into the PLR before the saccade begins (47), consistent with presaccadic processes. By accelerating the constriction of the aperture for the target of an upcoming saccade, attentional modulation of the PLR could improve the efficiency of visual scanning by adapting the pupil across luminance gradients found in natural vision. Of course, any advantage in scanning efficiency is theoretical and, if empirically observable at all, may be quite small (47).

**Figure 3:**
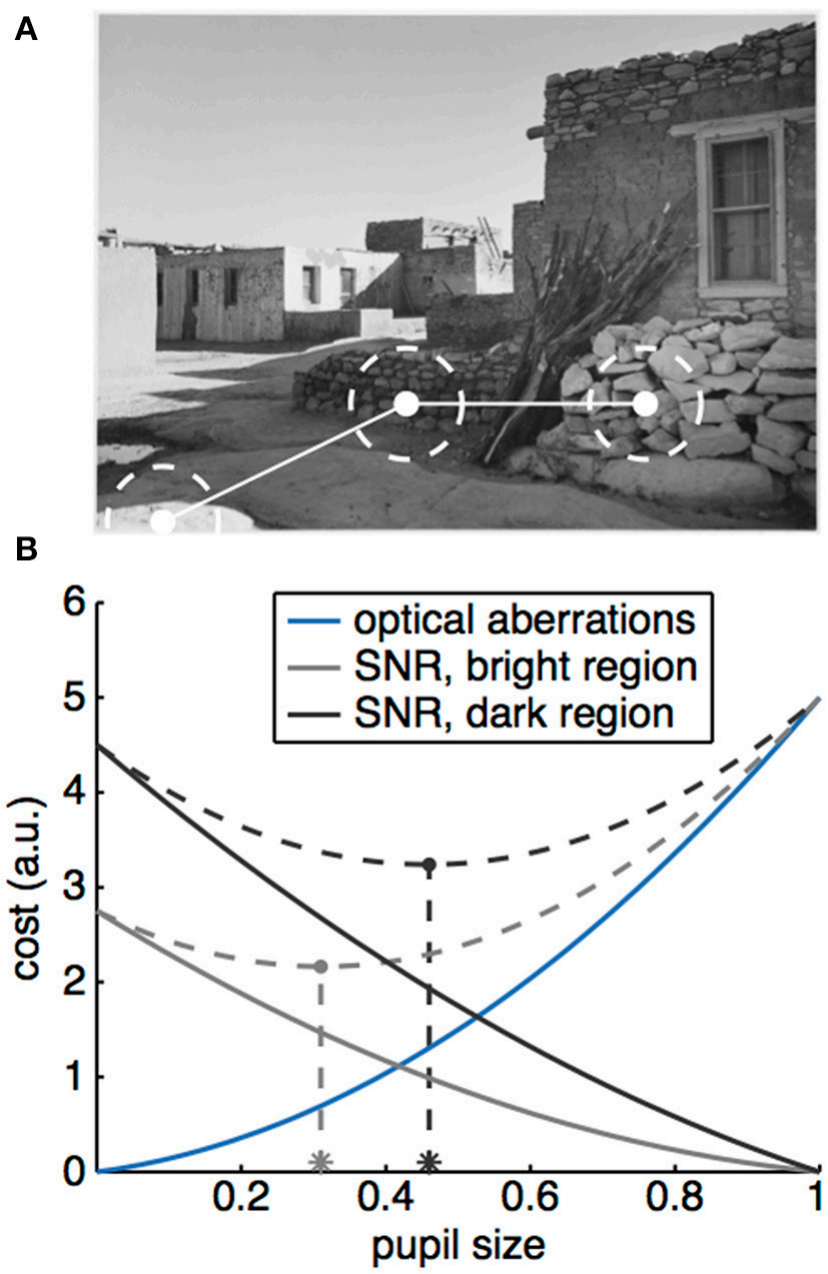
A potential role for the PLR in optimizing acuity in natural vision. **(A)** Natural scenes have luminance gradients, such that successive saccades can target regions with very different brightness (Image: Ansel Adams, “Acoma Pueblo. [National Historic Landmark, New Mexico],” U.S. National Archives, identifier #519836). **(B)** Cartoon illustrating how a tradeoff between signal to noise and optical aberrations could produce different optimal pupil sizes for different luminance regions. The effect of optical aberrations on vision (costs, solid blue line) increase as a function of pupil size. A larger pupil also increases the amount of light passing through, which means that the signal to noise ratio (SNR) would also increase as a function of pupil size, producing a decreasing cost for vision as a function of pupil size (solid gray and black lines). Because luminance varies across the scene, different regions would have different intrinsic signal levels, which would interact with pupil size to determine the costs (compare solid gray and black lines). To find the optimal pupil size across these two conditions, we can calculate the total costs due to both SNR and optical aberrations (dotted gray lines), then find the pupil size that minimizes these total costs (asterisks).

An alternative, and perhaps complimentary, hypothesis is that attentional modulations of the PLR may play a role in optimizing visual acuity across light intensities (4, 46). In this view, attentional modulations of the PLR may act to optimize visual acuity for the attended stimulus. This is because decreasing pupil size both limits the light hitting the retina (63), and improves visual acuity by reducing various optical aberrations (3–6). As there is background noise in our photoreceptor output (64, 65), there is a natural tradeoff, gated by the pupil, between visual acuity and the signal to noise ratio of the selected visual signal (66). Decreasing incoming drive by decreasing pupil size could bury a visual signal in the noise floor—unless that visual signal is sufficiently bright. By allowing attention to enhance the pupil constriction evoked by bright signals—beyond what would be evoked by the stimulus if attention was directed elsewhere—the eye could take advantage of the greater acuity that is possible when the incoming signal is brighter (Figure 3).

An important caveat to this argument is that attentional enhancements of the PLR likely have only subtle, small effects on visual acuity. These modulations enhance pupil constriction on the order of a tenth of a millimeter, which would produce negligible changes in acuity for eyes with normal or corrected-to-normal vision [though changes in acuity would certainly be larger in eyes with refractive errors; (3)]. Nevertheless, it remains unclear whether such small changes in pupil size would be sufficient to produce substantive changes in visual acuity during natural vision in primates today.

This question of magnitude is important because prefrontal control over a brainstem reflex requires long-range connections that seem costly to evolve and maintain. Shouldn't they confer some substantive benefit in order to have been selected for by evolution? It is important to note that a substantive benefit to perception in primates today is not necessary for either evolving attentional modulations of the pupil light reflex or for these modulations to have a function today. First, it is plausible that attentional modulations of the PLR are an exaptation (67)—a byproduct of evolution that was not selected for directly. In this view, these modulations could have evolved via a noisy selection process operating on some other, related competency, such as for prefrontal control over other brainstem circuits. Once evolved, exaptations nevertheless can be co-opted to serve some function, such as improving visual acuity during natural vision in the myopic eye. Second and alternatively, it is possible that attentional modulations of the pupil light response are vestigial: a competency that was selected for when it did confer a substantive benefit and maintained because it does not substantially hinder fitness. If the frequency of refractive errors has reduced over primate evolution, then it is possible that some ancestral nervous system evolved attentional modulations of the pupil light reflex at a time when they did provide substantive perceptual benefits, though these benefits have become smaller over time as the primate eye became increasingly emmetropic. Differentiating between these possibilities will require comparative studies. However, in either case observing attentional modulations of the pupil in the primate eye today does not imply or require that these modulations were selected for directly. It may imply that they did not hinder fitness enough to be selected against—perhaps because they work synergistically, rather than competitively, with other mechanisms for light adaptation or for improving visual acuity with attention.

## Cognition and Pupil Size Under Constant Luminance

In addition to modulating the PLR, covertly reorienting selective attention could also cause a transient pupil constriction. A small pupil constriction is observed following subtle equiluminant changes in stimuli, including changes in color, spatial frequency, structure, and motion (68–71). [This is in contrast to an arousing or alerting response to a highly salient stimulus, which produces pupil dilation, rather than constriction (72, 73)] However, these same subtle changes in stimuli also transiently capture and reorient selective visual attention (74, 75). These reorienting pupil constrictions depend on visual processing in the cortex: they are not observed in cortical blindness (68), where the visual signals in cortex are significantly reduced (76). Of course, reorienting pupil constrictions are quite transient—decaying within about 2 s after the evoking stimulus—but they could still permit a transient improvement in acuity, locked to the moment when a change in stimulus structure is capturing selective, spatial attention. Of course, future work is necessary to determine whether this pupil onset response is specifically related to reorienting selective visual attention toward a stimulus on a display, or if it is instead mediated by other mechanisms, such as a generalized alerting or arousal response.

Other cognitive processes produce sustained decreases in pupil size. For example, during a learning task, commitments to a behavioral policy are associated with sustained pupil constriction (11). Similarly, the pupil under constant luminance is smaller on trials following both errors of task performance and task conflict in a well-learned task (77). Together, these results suggest that pupil size may be tonically smaller when humans and other primates are committed to executing a well-learned behavioral task, rather than learning about a changing environment (11, 17, 78) or struggling to perform a difficult task (1, 9, 79).

Learning and task difficulty are not the only mental processes associated with *larger* pupil sizes. Instead, the pupil size increases with a diversity of cognitive processes including surprise (11, 12), motivation (80), emotion (81, 82), exploration (17), and many other cognitive processes that have been reviewed extensively elsewhere (9, 83). One interpretation of these effects is that the pupil simply increases in size with autonomic arousal—that modulations of autonomic arousal are some final common outcome of all of these cognitive processes (77). Indeed, pupil size covaries with other measures of autonomic arousal, including changes in skin conductance (79, 81) and activity in the locus coeruleus (84). Another interpretation of these results is that pupil size is larger any time a behavioral change is needed (i.e., when a surprising or arousing experience suggests that it is important to adapt behavior). In this view, pupil size under constant luminance and related cognitive or neural processes may track the balance between behavioral stability and flexibility (85–90). There is certainly some evidence in favor of the view that pupil size predicts changes in core components of flexibility, including behavioral variability (14, 77, 91) and learning (11, 17, 78). Moreover, neurons in the dorsal anterior cingulate, a part of the brain thought to be responsible for regulating the balance between stability and flexibility (88, 92–94) also encode information about or predict changes in pupil size (29, 77, 84, 95, 96). However, future work is necessary to determine whether there are pupil-linked changes in the behavioral and neural mechanisms that support flexibility and/or stability [e.g., (97, 98)].

In many studies, pupil size under constant luminance is used as a peripheral index of autonomic arousal (79, 81), noradrenergic tone (12, 91, 99–101), control states (77, 99), or changes in cortical processing (77, 102–105). Because of its utility in these applications, pupil size can be implicitly treated as a by-product of the process of interest, rather than a motoric consequence of these processes. However, it is also possible that these modulations of pupil size have some adaptive function in their own right. To address this possibility, we will next consider what effect changes in pupil size might have on information processing via examining how the aperture has been used in photography.

### The Photographer's Aperture

The optics of a camera and the human eye are certainly not the same. For example, the camera is not foveal, and modern camera lenses are corrected for many of the optical aberrations that plague the eye. However, we can still build an intuition for the functional consequences of changing pupil size by looking at historical photographs. This is because we, as viewers, operate on these photographs, much as we operate on the visual world around us. We decide where to saccade within these images based on some combination of the visual salience within the image and our top down goals or beliefs about what is important (21). The power of the photographer is to change how we view the veridical world—by shaping how the viewer perceives and interacts with the visual scene (7, 106, 107). Our suggestion here is that the brain operates the aperture of our eye to just such an end.

In the introduction, we discussed how larger apertures produce softer-focus images by increasing optical aberrations. This occurs because large apertures allow greater scattering of photons from adjacent sources. This is more pronounced when the plane of focus is even slightly misaligned with the sensors [i.e., the photographers' film or our eye's photoreceptors; (3)]. Of course, camera optics have improved substantially since the technology was first developed in the early nineteenth century and modern digital cameras often eliminate these aberrations in software. This means that today, photographers predominantly adjust their aperture to set the depth of field of a photograph. A large aperture produces a narrow depth of field, where much of the scene is out of focus. A small aperture, conversely, produces a deep depth of field, where the fine detail is preserved across a range of distances. However, in historical photographic images, we can still see how photographers adjusted the aperture in order to enhance or eliminate optical aberrations in order to achieve different goals over time.

**Figure 4:**
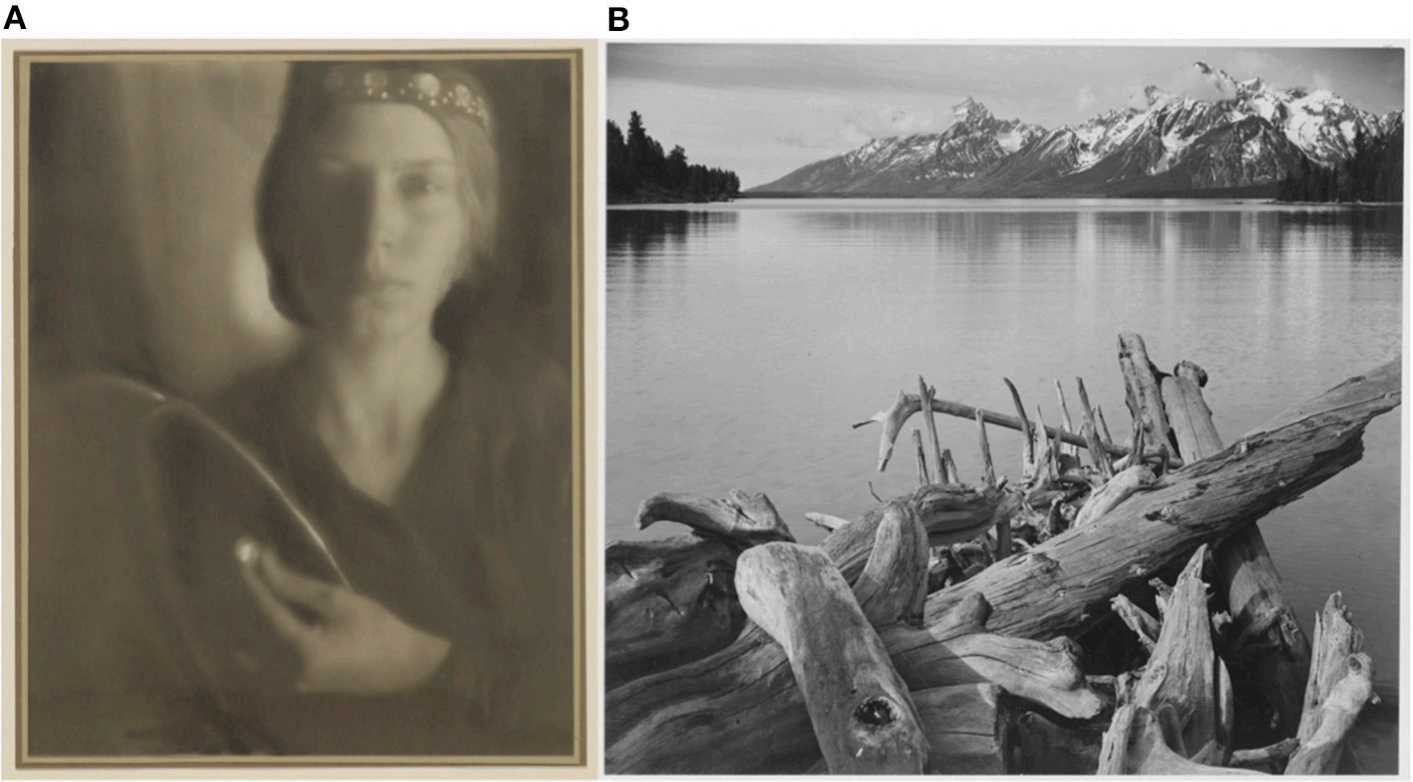
Example images from the Pictorialist and Purist photographic traditions. **(A)** “The Firefly,” A photograph in the Pictorialist style by George Seeley, 1911. (Source: Getty Museum, identifier: #84.XM.163.1). Note the soft focus and lack of high spatial frequency detail. **(B)** A photograph in the Purist style by Ansel Adams. “Jackson Lake, with Teton Ridge in the background.” Taken for the National Park Service, circa 1933–1942. Note the increase in fine, high spatial frequency detail. (Source: U.S. National Archives, identifier: #519909).

A large aperture produces a soft focus. That is, it reduces the fine, high-spatial frequency detail in the image, emphasizing larger forms at the expense of detail (Figure 4A). This type of aesthetic was exemplified in the images produced by “Pictorialist” photographers in the late nineteenth and early twentieth centuries (8, 108). Pictorialism was perhaps the first stylistic tradition in fine art photography, marking the transition of the camera from a mechanical device to a medium for artistic expression (106, 108). To separate this new form of photography from other, more technical uses, Pictorialists such as Alfred Horsley Hinton (106) sought to produce images that went beyond the “faithful and perfect delineation […] toward which Science and mechanics have striven in photography” (p. 5). Instead, Hinton described his goal to produce an image that captured the impression of a scene, noting “if the impression made upon me by the original scene was a very powerful one, then most probably I should have been unconscious of and be blind to petty details” (p. 7). Toward this end Hinton sought to capture “a general outline or by the portrayal of the chief items only” (p. 7) where “detail and crisp outlines [may be] intentionally subdued” (p. 8), and sharp lines are sacrificed for a depth of tones, an infinite gradation that makes objects appear to glow (106).

Pictorialist photographers used a range of techniques to produce these images. For example, in his 1917 manual on photography, Paul L. Anderson highlighted the benefits of selecting “a lens possessing all possible errors,” which gives, “as a result of its optical defects, a very soft and pleasing quality of definition” (p. 37). Anderson also described how the photographer could enhance these effects: “by the use of an aperture larger than normal it is possible to obtain greater diffusion, thus aiding in the suggestion of mystery, a suggestion which is of importance in any work of art” (p. 52). Many pictorialist photographers continued to work with large apertures and soft-focus lenses, long after the development of lenses corrected for various optical aberrations. This was particularly pronounced in Hollywood, where photographers and cinematographers continued to produce Pictorialism-inspired images for several decades after aberration-corrected lenses, “anastigmat” lenses, had been developed in the 1890's (7, 8, 109). These photographers produced the iconic images that we think of when we imagine female Hollywood icons the 1920's and 30's (8).

Starting in the 1920's and 1930's, however, many photographers began experimenting with precisely the opposite choice: using small apertures to produce images that engaged the viewer with detail. This became the Purist or objectivist photography movement (109). At the forefront of movement was Ansel Adams, perhaps the best-known American landscape photographer (Figure 4B). To achieve his engulfing views of the natural world, Adams used the smallest apertures that were available. This choice of aperture was so central to his process that he later formed a gallery and working group in Oakland, California under precisely this name [Group f/64; (109)]. Another member of this group was Edward Weston, a major master of 20th century photography. Weston used precisely the same techniques—long exposures with the smallest possible apertures—to produce richly detailed and absorbing images. In a 1930 essay declaring his disdain for the Pictorialists (“if they had no camera [they] would be third rate, or worse, painters”), Weston passionately described what he felt to be the true best-use of photography (107). The camera can “enable one to see through the eye, augmenting the eye, seeing more than the eye sees, exaggerating details, recording surfaces, textures that the human hand could not render with the most skill and labor.” The photograph “contains no lines in the painter's sense, but is entirely made up of tiny particles. The extreme fineness of these particles gives a special tension to the image, and when that tension is destroyed the integrity of the photograph is destroyed.”

The transition from Pictorialism to Purism in photography is clearly a far cry from what our pupils are doing as they transition from dilation and constriction. For example, the photographic apertures used to produce Figures 4A,B would have differed in size by more than the physiological range of the pupil—and certainly more than any cognitive modulation of the pupil. But, perhaps these images have value as a caricature of the effects of pupil size on perception: these images enhance and emphasize the effects of aperture size on our image of the world around us. Moreover, by looking at how we interact with these images as a viewer—and by thinking about what information is coded in the high spatial frequency channels that were enhanced in purism and discarded in pictorialism—we might be able to gain some insights into what, if any, function cognitive modulations of the pupil could serve.

### Possible Perceptual and Oculomotor Effects of Pupil Size

Small pupils would bias visual representations toward the purist tradition—emphasizing the fine detail and high spatial frequency information of the visual world. In a sense then, the pupil constriction caused by attention-capturing changes in stimuli (68–71) would mirror the known effects of attention on visual acuity (110) and contrast sensitivity (111). This is because, by decreasing defocus, smaller pupils necessarily increase visual acuity and sensitivity for the fine-grained contrast gradations typical of attention tasks (3–6). (Of course, larger pupils might increase contrast sensitivity for larger spatial scales, because they could increase the signal to noise ratio of vision by allowing more light to hit the retina).

Although there are clear parallels between the pupil constriction observed at attentional reorienting and the effects of pupil constriction on vision, several caveats must be noted. First, the perceptual consequences of attention and pupil size differ in space. *Selective* visual attention only improves contrast sensitivity and visual acuity only for a selected region in visual space (110–113). Conversely, any change in pupil size is necessarily a *global* effect. Thus, pupil constriction at attentional capture can provide a global compliment to ongoing, local perceptual processes. Second, the perceptual consequences of attention and pupil size also differ in magnitude. Attention improves visual acuity on the order of several arc minutes (114). For individuals with normal (20/20) vision, the change in pupil size that would be required to produce the equivalent change in visual acuity would be larger than the physiological range of the pupil (3). Of course, the perceptual effects of small reductions in pupil diameter can produce arc minute changes in visual acuity in myopic or astigmatic individuals (3). Thus, effects of pupil constriction on vision complement and may work synergistically with other known effects of attention on vision.

What consequences would increasing the fine detail in a visual representation have for gaze and perception? In natural vision, high spatial frequency information scales with proximity, such that closer objects and features contain finer details (115). Given that this information carries forward through the visual system to preferentially attract gaze (20, 21, 116, 117), enhancing the representation of this information by any means—including decreasing pupil size—could help to bias perception and gaze toward nearby objects, rather than distant ones. Indeed, pupil constriction is a fundamental component of the near response. That is, when we do focus on a nearby object, a triad of oculomotor effects occurs: the eyes converge, the lenses accommodate, and the pupils constrict (118, 119). Of course, future work is necessary to determine whether changes in pupil size on the order of these cognitive influences can produce perceptual biases of sufficient magnitude to modulate gaze.

**Figure 5:**
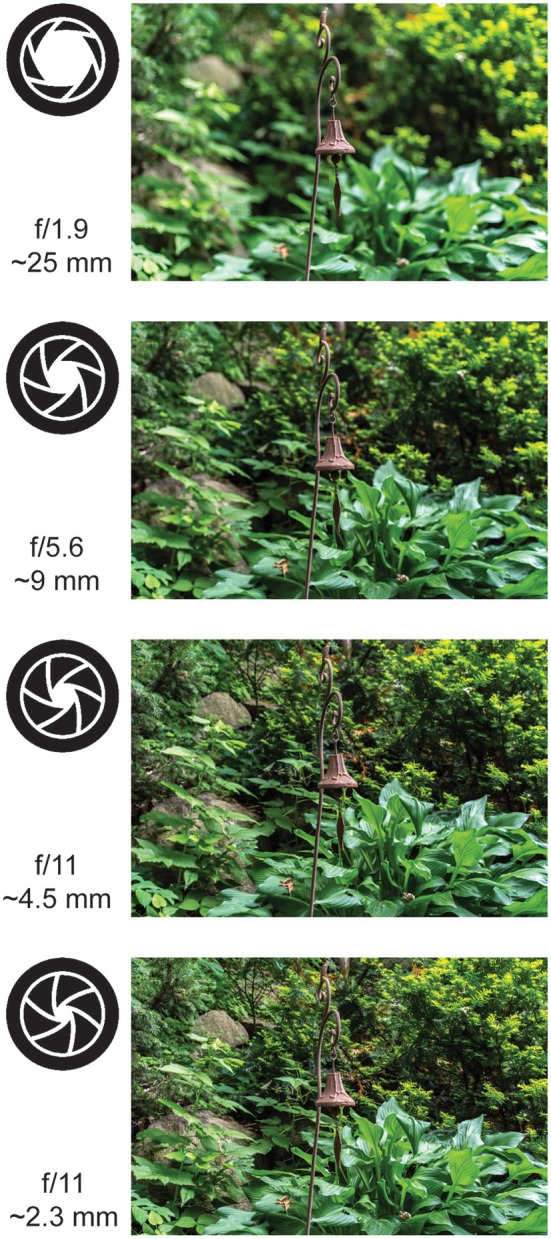
The effects of aperture size on depth of field. (Top to bottom) The effects of decreasing aperture size on defocus in three dimensions in a modern digital camera. The large aperture increases the sense of depth in the top photographs, but the small aperture increases the high spatial frequency information in the bottom photographs. Note that the range of aperture sizes used here is larger than the physiological range of the pupil (which is only 2–8mm) and these images were corrected for optical aberrations by processing in the camera (Photo credit: Boris Oicherman).

The idea that the pupil constriction component of the pupil near response could function to focus gaze on nearby objects seems at odds with the observation that small apertures are used to produce a deeper depth of field in modern photography. A small aperture allows the region of focus to extend further both toward and away from the viewer (Figure 5). This implies that there would be more high spatial frequency information when pupils are small across both nearby and distant locations—not just in the current plane of focus. Of course, this would not change the fact that nearby objects contain more high-spatial frequency information, so increasing depth of field could still bias gaze toward nearby objects. It is also important to note that while there is substantial evidence that decreasing pupil size (< 4 or 5 mm) does narrow the eye's depth-of-field (120, 121), the effects of pupil size on depth-of-field in the eye are not necessarily as straightforward as they are in a modern digital camera. For example, depth-of-field in our visual systems is also substantially affected by optical aberrations that are eliminated in these cameras, there are modulatory effects of both neural and retinal processing (121), and there is evidence that increasing pupil size (e.g., from 4 to 6 mm) can narrow, rather than deepen depth-of-field (121). Moreover, because blur is important for estimating depth (122, 123), it is possible that the function of any deepened depth of field with small pupil size is to reduce this depth information when it is unnecessary—such as in near-work tasks. Ultimately, future work is needed to determine how pupil size affects perception and gaze in three dimensional environments.

In higher level vision—visual representations of objects or social partners—high spatial frequency information often carries a disproportionate amount of information about the identity of that visual target (124–127). Moreover, there is evidence that changing goals could change the spatial frequency information that we prioritize for processing (128, 129). For example, human observers tend to rely on high spatial frequency information to discriminate facial identities (125, 127, 130) or to differentiate objects within broader conceptual categories [i.e., breeds of dogs (125) or the identity of a specific toy (126)]. Enhancing high spatial frequency information could help the viewer to individuate objects, perhaps even along task-relevant dimensions. Of course, this is a strong prediction—that decreasing pupil size would increase individuation of faces and objects—and empirical studies are needed to determine whether cognitive modulations of pupil size are of sufficient magnitude to produce this kind of change.

If decreasing pupil size draws gaze to nearby objects, increasing pupil size might have the opposite effect reducing the drive to look at proximate objects by eliminating the high-spatial frequency information that may partially drive this bias (20, 21, 115–117). It is intriguing to note that this could focus gaze on regions with large changes in contrast across low spatial frequencies for two reasons. First, it would reduce the encoding (and thus salience) of competing high spatial frequency information. Second, it could potentially increase contrast sensitivity at low spatial frequencies by allowing more light into the eye. By allowing more photons to hit the retina, large pupils could increase the signal-to-noise ratio of vision—effectively increasing the perception of contrast at any spatial scale that is larger than the scale of dilation-induced defocus. Additional work is necessary to determine how pupil size affects contrast sensitivity at various spatial scales. However, there is some evidence for the view that pharmacological perturbations that increase pupil size do bias gaze toward regions where contrast varies substantially across large regions in space (131, 132), though it remains unclear whether these effects are mediated by changes in pupil size or by changes in neural activity (14, 29).

Other perceptual consequences of reducing high spatial frequency information may be to bias perception toward global, categorical, and configural properties of the visual world (133, 134). We know that low spatial frequency information is sufficient, and indeed more useful than high spatial frequency information, in rapidly perceiving the gist of a scene (135). This implies that observers with larger pupils might focus more on global properties of a scene, rather than the specific details. In object perception, pupil dilation might decrease information about object or face identity in favor of type, mimicking the effects of disrupting high spatial frequency information in images (124–127). This could make it easier to represent a figure according to more abstract classes—such as whether an object is a car or an animal—rather than according to fine grain distinctions between different animals (136). Finally, large pupils could also aid in estimating the three dimensional configuration of a scene—that is, the distance between ourselves and some salient cue. This is because blur is a salient depth cue, useful for estimating distances in three dimensions (122, 123), and it also increases as the depth of field decreases with large pupil sizes (Figure 5).

Given the link between pupil size and autonomic arousal, an increase in configural processing with large pupil sizes could certainly be adaptive. Much as sympathetic arousal quickens heart rate and shifts blood flow to skeletal muscles, perhaps it also changes our visual filter in order to more rapidly and accurate differentiate between trees and tigers and estimate their distance from ourselves, without regard for the texture or individual identity of either. This could also help with generalizing learned associations to members of a broader class. For example, if your previous experiences with tigers have been particularly arousing, perhaps it is best that those memories generalize across all big cats. It is probably a waste of limited neural resources to even represent the specific details of any given tiger!

Of course, it is also possible that cognitive modulations of the pupil are not large enough to produce any substantial perceptual change, given the modern primate eye. This is an empirical question that can be addressed psychophysically, either through combining pharmacological dilation with artificial pupils or through filtering images to match the putative effects of plausible changes in pupil size. However, even if cognitive modulations of the pupil are not sufficiently large to produce perceptual changes in the normal primate eye today, this does not preclude the possibility that they either evolved in an ancestral eye, where they did produce adaptive perceptual changes. Their continued existence today may simply imply that they did not hinder fitness enough to be selected against—perhaps because they work synergistically, rather than competitively, with other mechanisms for biasing perceptual processing according to pupil-linked goals or brain states.

## Directions for Future Research on Perception, Gaze, and Pupil Size

Much of the above is a speculative juxtaposition of the known optical consequences of pupil size and the effects of various image manipulations on natural image viewing. In particular, we have noted that more work is needed to determine the precise magnitude of any effect of pupil size on perception and any perceptual effects on gaze and behavior. Although pupil size necessarily gates spatial frequency and blur information, to our knowledge, few studies have looked at how pupil size influences gaze and visual perception. In part, this is because naturally-occurring fluctuations in pupil size are inexorably linked with changes in autonomic arousal (79, 81), noradrenergic tone (12, 91, 99–101), control states (77, 99), and cortical processing (77, 102–105). Any of these processes could produce changes in gaze, visual processing, or task performance via mechanisms other than pupil size.

Of course, the converse is also true: without knowing the effects of pupil size on gaze, visual processing, and task performance, we cannot ascribe behavioral correlates of pupil size to changes in arousal, norepinephrine, control states, or cortical processing. This is because any behavioral correlates of pupil size—even those that seem deeply cognitive *primafacie*—could be due to the effects of pupil size on perception, rather than a latent state that is indexed by pupil size.

Changes in low-level perceptual cues can have substantial consequences for higher-order cognitive processes. For example, there is certainly some evidence that the spatial frequency of vision is consequential for higher order cognitive processes, including as memory and decision-making. One thoughtful study reported that small pupil size at encoding was associated with better recognition memory for objects (137). Does this mean that pupil-linked mechanisms such as arousal, norepinephrine, control states, or cortical processing underlie recognition memory? There are two pieces of evidence that suggest otherwise. First, this study also noted that subjects with smaller pupils also made more frequent, shorter direction fixations when viewing the objects. These patterns of gaze predicted future recognition memory just as well as pupil size did. We know from other studies that these gaze patterns mimic the effects of increasing high spatial frequency information in an image (116) and that high spatial frequency information is important for object recognition (138). Thus, it is entirely possible that the increase in recognition memory in this study was mediated by a change in the way the pupil filtered the visual world. Second, if pupil size indexed some brain state that was optimized for memory encoding, it should have the same relationship with recognition memory, regardless of what kind of information was being encoded. Yet, small pupils at encoding may only predict better recognition memory for objects (137). Small pupils are associated with *poorer* recognition for faces (139). This is striking because low—not high—spatial frequency information is essential for encoding faces (140–142). Thus, during object encoding, smaller pupils would preserve the high spatial frequency information that is important for object recognition—drawing attention *toward* the critical stimulus dimensions. However, during face encoding, smaller pupils would preserve the high spatial frequency information that competes with the important low spatial frequency cues—in this case, drawing attention *away* from the critical stimulus dimensions.

**Figure 6:**
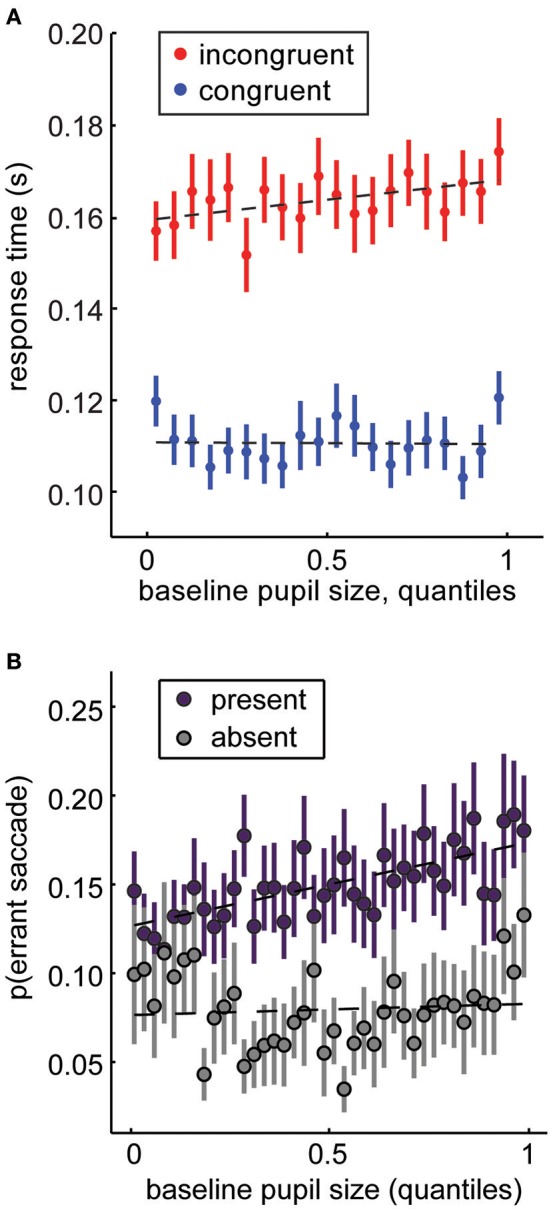
Baseline pupil size under constant luminance predicts changes in attentional priorities. **(A)** In the distraction task (Figure 1A), large, salient distractors are presented in conflict with a rewarded target. Monkeys are faster for congruent distractors and slower for incongruent distractors (Figure 1C). Increasing pupil size magnifies these effects: attention is more affected by the distractors when pupil size is large. **(B)** In the same task, we can also measure the probability that monkeys would make an “errant saccade” to a task-irrelevant distractor, rather than a rewarded target (these trials were excluded from analysis in **A**). Errant saccade likelihood increases as a function of pupil size at fixation. Panel **(A)** is modified from Ebitz et al. (14) and is reproduced under a Creative Commons Attribution license. Panel **(B)** is modified from Ebitz and Platt (77) with permission from Cell Press and Elsevier.

Mnemonic encoding is not the only cognitive process that can be gated by perception or the effects of perception on gaze and attention. For example, we know that gaze is sufficient to shape economic (143) and social (144) decision-making. Perhaps this occurs because gaze gates value signals in higher order decision-making regions (145). This suggests that changes in fixations patterns propagate through decision processes to shape behavior. By systematically reducing the high spatial frequency information, pupil size could bias gaze, and therefore decisions away from high spatial frequency targets. For example, we previously found that pupil size predicted decisions to look at large (~15° visual angle) images of conspecific faces, rather than small (< 1°) rewarded targets [Figures 6A,B; (14, 77)]. We interpreted this as a change in monkeys' susceptibility to distraction, but it is entirely possible that this susceptibility was mediated by a change in the monkeys' percept of the visual display. Perhaps their larger pupils simply deemphasized the visual salience of the small rewarded target (29). In a natural environment, this shift in perception could mean the difference between decisions to forage at a local patch (which is necessarily richer in high-spatial frequency information by virtue of its proximity) or decisions to explore more distant opportunities.

Much more work is necessary to establish what effect pupil size has on gaze and attention, much less on higher order cognitive processes like memory and decision-making. This is a substantial need because, as we have just illustrated, it can be tempting to think of pupil size as an *index* of a latent brain state, but it remains possible that pupil size could *cause* changes in perception that then influence cognition via shaping attention or gaze.

Because of these potential confounds, building a taxonomy of the direct behavioral effects of pupil size is an essential precondition for the use of the pupil as an index of any cognitive or neural state. First, it is important to determine how spatial frequency information changes across pupil size. One promising approach might be to have human subjects report their percepts of ambiguous images, that contain differing objects or scenes in parametrically varied frequency channels (128). This would allow a quantitative description how pupil size sets the spatial frequencies that are prioritized for processing. Second, it is necessary to determine whether any neural and/or behavioral correlates of pupil-linked states can be replicated by filtering the display to enhance or suppress these frequency channels. An alternative and perhaps complementary approach might be to dilate the pupils with mydriatic agents (such as the tropicamide and the phenylephrine), then use an artificial “pupil” to determine whether manipulating pupil size was, by itself, sufficient to replicate any behavioral effects (66). Addressing these two questions will be essential for both understanding the perceptual consequences of pupil size and for establishing the circumstances in which pupil size does simply index a latent mental state.

## Discussion

Our central hypothesis is that cognitive modulations of the pupil may be functional, rather than epiphenomenal. To illustrate this perspective, we first discussed the attentional modulation of the pupil light reflex. Previously, we reported that these modulations can be qualitatively reproduced by electrically stimulating part of the prefrontal cortex involved in directing visual spatial attention (28). This suggests that the brain somehow evolved prefrontal control over a brainstem pupil reflex—a motif that seems costly to evolve and/or maintain had it not conferred some adaptive benefit. We highlighted two potential functions this descending control might have, but cautioned that more work is necessary to determine the magnitude of these effects on visual acuity, sensitivity, and light adaptation.

Next, we posited that the pupil may act to filter the visual world—to emphasize some visual features while suppressing others. We highlighted high spatial frequency information as the primary type of information that would be preserved when pupils are small, but suppressed when they are large. This is because optical aberrations—which cause blur and defocus at fine spatial scales—increase as the pupil gets larger. This means that when the pupil is large, the visual world is rendered with a Pictorialist brush: defocus and blur are maximal. Conversely, when the pupil is small, the visual world is rendered in the Purist tradition: rich with detailed, high spatial frequency information.

We have argued that blur and defocus are ideal in circumstances where processing larger forms—e.g., the class of an object, the gist of a scene—is most beneficial. It seems to us that these circumstances are precisely the ones in which pupil size is at its largest—the circumstances where rapid decision-making and generalization across classes are perhaps the most useful for our survival. High spatial frequency information, conversely, is the currency of visual attention, where the selective processing of this fine grained information is critical for individuating targets by recognizing differences in the fine details that differ between them.

## Author Contributions

RE and TM formulated the hypothesis and wrote the manuscript.

### Conflict of Interest Statement

The authors declare that the research was conducted in the absence of any commercial or financial relationships that could be construed as a potential conflict of interest.
